# UAV-Based Smart Educational Mechatronics System Using a MoCap Laboratory and Hardware-in-the-Loop †

**DOI:** 10.3390/s22155707

**Published:** 2022-07-30

**Authors:** Luis F. Luque-Vega, Emmanuel Lopez-Neri, Carlos A. Arellano-Muro, Luis E. González-Jiménez, Jawhar Ghommam, Maarouf Saad, Rocío Carrasco-Navarro, Riemann Ruíz-Cruz, Héctor A. Guerrero-Osuna

**Affiliations:** 1 Centro de Investigación, Innovación y Desarrollo Tecnológico CIIDETEC-UVM, Universidad del Valle de México, Tlaquepaque 45604, Jalisco, Mexico; luis.luque@uvmnet.edu (L.F.L.-V.); emmanuel.lopezne@uvmnet.edu (E.L.-N.); 2 Research Laboratory on Optimal Design, Devices and Advanced Materials—OPTIMA, Department of Mathematics and Physics, ITESO, Tlaquepaque 45604, Jalisco, Mexico; carellanomuro@iteso.mx (C.A.A.-M.); rociocarrasco@iteso.mx (R.C.-N.); riemannruiz@iteso.mx (R.R.-C.); 3 Department of Electronics, Systems and Informatics, ITESO, Tlaquepaque 45604, Jalisco, Mexico; luisgonzalez@iteso.mx; 4 Department of Electrical and Computer Engineering, College of Engineering, Sultan Qaboos University, Al-Khod, Muscat 123, Oman; jawher@squ.edu.om; 5 Department of Electrical Engineering, École de Technologie Supérieure, Montreal, QC H3C 1K3, Canada; maarouf.saad@etsmtl.ca; 6 Posgrado en Ingeniería y Tecnología Aplicada, Unidad Académica de Ingeniería Eléctrica, Universidad Autónoma de Zacatecas, Zacatecas 98000, Zacatecas, Mexico

**Keywords:** UAV, educational mechatronics, Industry 4.0

## Abstract

Within Industry 4.0, drones appear as intelligent devices that have brought a new range of innovative applications to the industrial sector. The required knowledge and skills to manage and appropriate these technological devices are not being developed in most universities. This paper presents an unmanned aerial vehicle (UAV)-based smart educational mechatronics system that makes use of a motion capture (MoCap) laboratory and hardware-in-the-loop (HIL) to teach UAV knowledge and skills, within the Educational Mechatronics Conceptual Framework (EMCF). The macro-process learning construction of the EMCF includes concrete, graphic, and abstract levels. The system comprises a DJI Phantom 4, a MoCap laboratory giving the drone location, a Simulink drone model, and an embedded system for performing the HIL simulation. The smart educational mechatronics system strengthens the assimilation of the UAV waypoint navigation concept and the capacity for drone flight since it permits the validation of the physical drone model and testing of the trajectory tracking control. Moreover, it opens up a new range of possibilities in terms of knowledge construction through best practices, activities, and tasks, enriching the university courses.

## 1. Introduction

The next era of the industrial revolution is a reality, and many companies are integrating the concepts of Industry 4.0 into their processes. Industry 4.0 proposes the digitalization of companies through Artificial Intelligence (AI) and the Internet of Things (IoT). Incorporating new technologies, from Information and Communications Technology (ICT), within the industrial environment has been changing business models as we know them. It is worth mentioning that the present work is based on [1], in which an unmanned aerial vehicle (UAV) flight instructional design for Industry 4.0 based on the framework of educational mechatronics is presented.

Recent years have witnessed a growth in the interest in identifying the different scenarios that Industry 4.0 brings due to the new challenges that appear on the horizon [2]. According to [3], the literature review identified four key components of Industry 4.0: cyber-physical systems, Internet of Things, Internet of Services, and smart factories. Within the framework of Industry 4.0, the innovative idea of including drones as part of the automation ecosystem emerges. Drones in Industry 4.0 begin to be seen as a solution for process improvement, so understanding the operation of a drone is becoming an essential skill for new applications in Industry 4.0. These devices require skilled operators to manage, configure, program, control, and provide maintenance. The first step toward the drone world is to know the UAV dynamics and its flight principles, as well as the hardware and software related to its use and control.

Drones, as an emerging technology, have been taught since 2013 [4]. Since then, there have been modifications to the k-12 drone curriculum to teach and increase the science, technology, engineering, arts, and mathematics (STEAM) skills associated with the use of this technology [5], mainly under the approach of problem-based learning (PBL), integrating drones as a teaching technology [6,7,8]. This approach aims for the student to understand the operation from abstract concepts such as formulas or mathematical models [9,10]. In addition, the learning process is a mechanical consequence of their assimilation. Hence, if students learn a solution for such mechanized learning patterns and problems arise in the real world, this solution does not correspond to the pattern learned. Frustration occurs in the student and consequently does not allow adaptation to the rapid changes required by Industry 4.0, such as new knowledge, skills, and abilities for the new jobs brought by Industry 4.0. Therefore, higher education institutions need to establish strategies to generate the human resources that support the demand of this industry [11].

Amongst these necessary emerging abilities, hardware-in-the-loop (HIL) simulation plays an important role during the development of new products and services as it accelerates their design and testing phases [12] in engineering fields such as power electronics [13], autonomous vehicles [14], and robotics. Therefore, its integration with educational methodologies has been studied for teaching control design [15], automation engineering [16], power electronics [17], mechatronics [18], and other technological topics. The framework proposed by this type of simulation represents great advantages to the industrial environment as it is easily integrated with extensively used concepts such as model-based design and the V-cycle. Furthermore, it is possible to develop a HIL simulation platform with limited economic resources [19]. Due to this, the HIL simulation concept is integrated within the proposal developed in the presented work.

However, the adaptation rate to reduce the gap between the new required industry knowledge, skills, and attitudes against current curricula from higher education institutions is very slow. The above is caused by the educative policies and the complexity of raising organizational changes in the higher education institutions’ operational structures. The current context brings an opportunity to develop a novel framework that allows the dynamics of higher education institutions to meet the speed of change in which technology evolves. This work proposes a UAV-based smart educational mechatronics system that uses a motion capture (MoCap) laboratory and HIL to teach the required knowledge and skills when working with UAVs. In particular, UAV knowledge includes topics such as flight dynamics, mathematical models, path planning, and control design, among others. Moreover, UAV skills involve spatial location, waypoint navigation, the ability to fly a drone, etc. The applied methodology is based on the Educational Mechatronics Conceptual Framework (EMCF), which is focused on ensuring the development of student learning to build different mechatronic concepts. This involves knowledge organization in a structured way but does not focus on the connections that allow the reconstruction of problems outside of what is usually found in the industry.

In particular, a case study in developing the mechatronic concept of drone navigation by waypoints is presented as it represents one of the significant challenges in robotics and autonomous vehicle research fields. The proposal is based on a motion capture system to retrieve the drone’s state during the pedagogical experiments’ development. The MoCap system permits obtaining measurements for the inertial position and attitude of the vehicle. Then, this information is used as input for the overall educational framework levels and its instructional design. Moreover, a HIL simulation is used to validate the physical UAV waypoint navigation. Compared to the reported scientific literature, the novelty of this work relies on a combination of educational tools such as the drone, MoCap, and HIL simulation to construct knowledge and skills in the students within the EMCF.

The rest of the document is organized as follows: Section 2 describes the EMCF. Then, the materials and methods applied during the proposed activities are defined in Section 3. After this, the proposed instructional design and its levels are described in Section 4. Finally, a discussion and the main conclusions on the results of the presented work are outlined in Section 5 and Section 6, respectively.

## 2. Educational Mechatronics Conceptual Framework

The EMCF aims to guide teachers on designing, implementing, and evaluating pedagogical activities to develop mechatronic thinking in students. The latter is understood as the capacity for designing and implementing production systems [20] under the principle of interdisciplinary collaboration. In addition, it is important to understand the concept of multidisciplinary provision of knowledge [21], in a flexible way [22,23], considering the high-level intelligence hierarchy as the backbone of the mechatronic system [24,25]. Educational mechatronics is intended to allow the student to understand abstract concepts based on which the applications we call mechatronics are built. Moreover, they will thus be able to face the speed of growth and exponential change of Industry 4.0, responding to the megatrends of the manufacturing industry and advanced manufacturing processes, focusing on the development, application, or integration of a set of enablers and technologies in order to generate impact [26].

**Figure 1:**
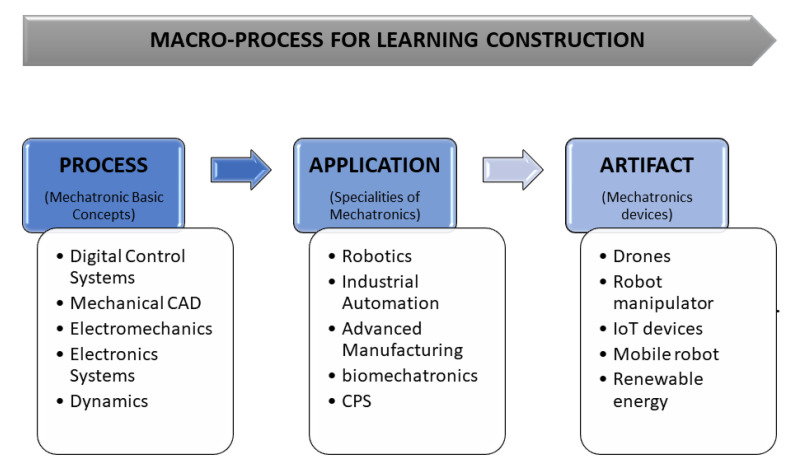
The mechatronics educational framework and disciplines.

The EMCF is structured into three reference perspectives, process, application, and artifact [27], as shown in Figure 1. The first perspective is oriented to mechatronics’ basic concepts as a process. The second perspective comprehends all the applications (sub-disciplines) from the basic mechatronics concepts. Moreover, finally, the artifact is oriented to obtain some artifacts related to the process and application construction. In summary, the EMCF is structured into three reference perspectives: process, application, and artifact [27].

**Figure 2:**
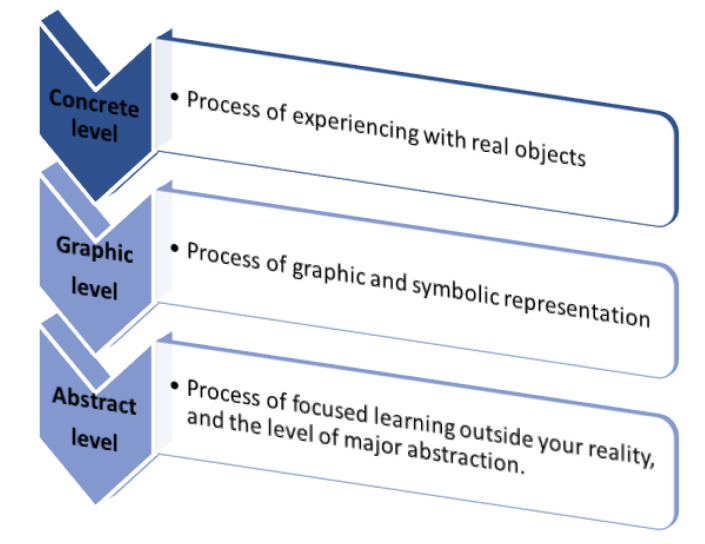
Educational Mechatronics Conceptual Framework: macro-process levels and processes involved in the construction of learning.

The macro-process learning construction of the EMCF is based on the structured teaching methodologies proposed by [28,29]. Figure 2 shows the three learning levels: concrete, graphic, and abstract. The first level involves the process of real object manipulation and experiences [30,31]. The second level relates the elements of reality (concrete level) to graphics or symbolic elements, enabling students to integrate this knowledge as a skill [32]. Finally, the third level represents the highest level of abstraction and focuses on learning outside of reality.

## 3. Materials and Methods

The materials and methods comprising the UAV-based smart educational mechatronics system are chosen based on the mechatronic prototypes and existing academic spaces at the Universidad del Valle de México: DJI Phantom 4, a MoCap laboratory giving the drone location, a Simulink drone model, and an electronic board for performing the HIL simulation. Moreover, the proposed instructional design is aligned with the EMCF.

### 3.1. DJI Phantom 4

**Figure 3:**
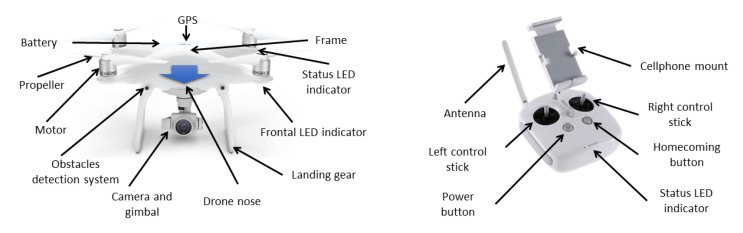
Drone in the MoCap system.

DJI Phantom 4 is a quadcopter equipped with a collision-avoidance system, called an Obstacle Sensing System, which uses two forward-facing cameras to detect obstacles as far as 49.5 ft (15 m) ahead of the drone. The drone comes mainly with a remote controller, camera, and gimbal (see Figure 3).

It is worth mentioning that drone flight phases involve takeoff, flight, and landing.

- Takeoff: this is the phase where the drone accelerates from zero speed to the speed necessary to rise to a certain altitude at which the takeoff is considered to have finished.
- Operational flight: in this phase, the drone can hover (hold a stationary position in the air) and maneuver in flight, where mixed movements to the left, right, forward, backward, up, and down are possible.
- Landing: this is the phase where the drone approaches the destination and the landing gear makes contact with the runway while decelerating its motors until reaching zero speed.

### 3.2. Motion Capture System

**Figure 4:**
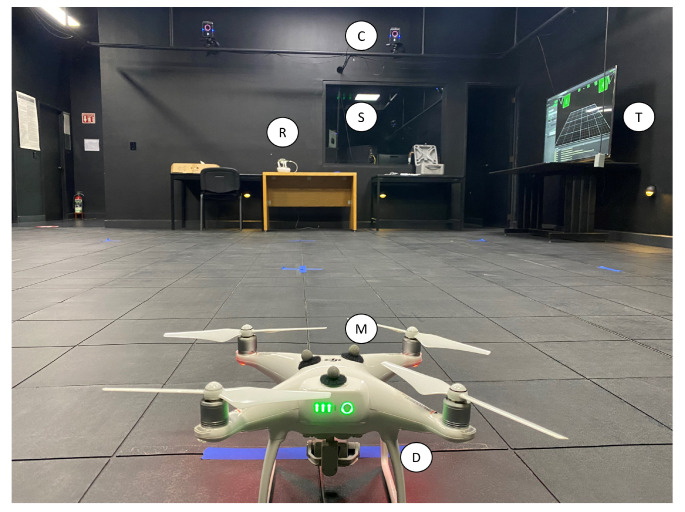
Components of the UAV-based smart educational mechatronics system: drone (D), markers (M), remote control (R), server computer (S), camera (C), and TV monitor (T).

The MoCap system installed in Universidad del Valle de México is shown in Figure 4; this is a market-based system that consists of the following elements:

- Eight Vantage V16 cameras contain a thermal sensor to detect changes in temperature that could affect the system status;
- Power over Ethernet (PoE) switch, where power and connectivity are through PoE plus a protocol by CISCO Systems^®^; an American-based multinational technology conglomerate corporation headquartered in San Jose, CA, USA.
- Lock Sync Box connects, integrates, and synchronizes the cameras through a PoE switch.
- Server computer with Vicon Tracker Software^®^  version 3.5.1  [33], which is a specialized software created by Vicon Motion Systems Ltd.^®^; an English-based corporation headquartered in Oxford, United Kingdom. This is used for tracking multiple objects, single-camera tracking, and real-time modeling, among others. In Addition, the server is equipped with the software Simulink^®^ version 2021b created by MathWorks; an American-based corporation headquartered in Natick, MA, USA. It is used for modeling, collecting, and analyzing drone variables data. Vicon Tracker ^®^ and Simulink^®^ softwares are compatible and fulfills several engineering needs.

**Figure 5:**
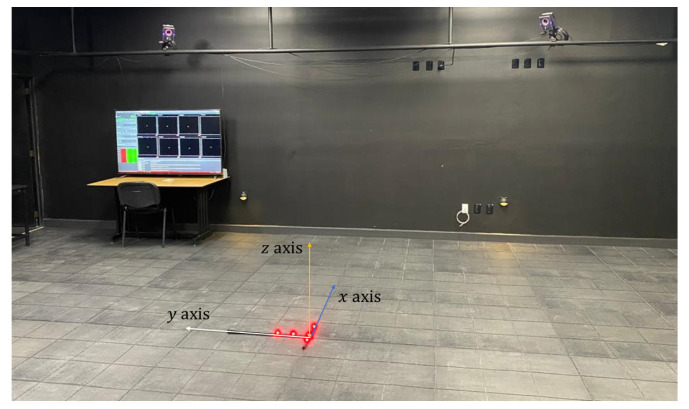
Origin setup of the MoCap system workspace.

To work with the MoCap system, first, it is necessary to locate all cameras properly in 3D space; then, calibration of the Vicon hardware must be done. To do so, turn on the PoE switch, the server computer, and open the Vicon Tracker program. Then, select the “SYSTEM” tab and select the eight cameras. Go to the “CALIBRATE” tab and click “START”. One person must take the active wand, turn it on with the solid red LEDs, and go to the MoCap system workspace, and then start moving the wand in different directions with different orientations in front of each camera. Once the process is finished, the Vicon Tracker software will send the calibration results; if everything is green, it does not indicate that the process was carried out satisfactorily; otherwise, it will have to be done again. Finally, the active wand must be placed where we want to establish the origin of the MoCap workspace (see Figure 5).

Next, to continue the setup to have the drone working in the MoCap system, the markers are attached to the drone frame, as shown in Figure 4, and an object representing the drone must be created using the Vicon Tracker software. Finally, the measurements from the MoCap system are collected with Simulink, a MATLAB-based graphical programming environment for modeling, simulating, and analyzing multidomain dynamical systems. In this case, the drone’s 2D positioning and orientation graphs and a 3D graph of its absolute position are displayed to the participant on the TV monitor. It is worth mentioning that the 50-inch TV monitor plays a key role when designing the instructional design based on the EMCF.

### 3.3. Simulation Model of the Quadrotor in Simulink

The quadrotor dynamical model is the result of analyzing the gyroscopic effects on the rigid structure of the multirotor due to the thrust forces generated by four rotating propellers. These propellers are attached to the axes of four brushless DC motors. The whole dynamics of the aerial robot involve two main reference frames, the earth-fixed frame and body-fixed frame, whose origins are located in the origin defined by the wand in the MoCap system (see Figure 5) and the center of mass of the quadrotor defined by the markers in the drone with an offset (see Figure 4), respectively.

The absolute pose of the quadrotor must be expressed in the earth frame, which is composed of its Euclidean 3D position $X_{E}={\left[x,y,z\right]}^{T}$ and its attitude $\Theta ={\left[\varphi ,\theta ,\phi \right]}^{T}$, which is represented by the Euler angles.

**Figure 6:**
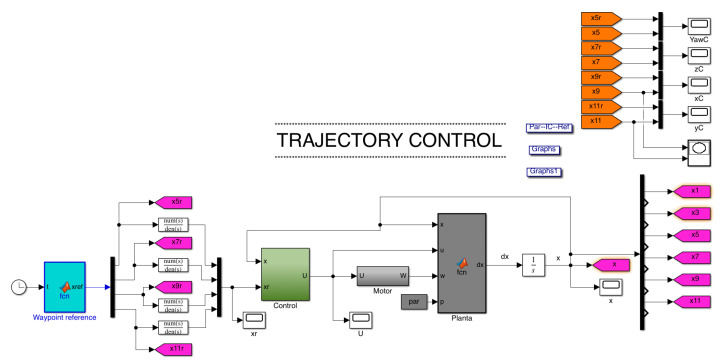
Quadrotor mathematical model in Simulink.

The dynamics of a quadrotor, in its state space form, can be defined by defining the state vector as $X={\left[x,\dot{x},y,\dot{y},z,\dot{z},\phi ,\dot{\phi },\theta ,\dot{\theta },\psi ,\dot{\psi }\right]}^{T}$, and it is described by the following differential equations:

(1) $$\begin{array}{ccc} {\dot{x}}_{1} & = & x_{2}\\ {\dot{x}}_{2} & = & \frac{U_{1}}{m}\left(S_{x_{7}}S_{x_{11}}+C_{x_{7}}S_{x_{9}}C_{x_{11}}\right)-A_{2}\\ {\dot{x}}_{3} & = & x_{4}\\ {\dot{x}}_{4} & = & \frac{U_{1}}{m}\left(-S_{x_{7}}C_{x_{11}}+C_{x_{7}}S_{x_{9}}S_{x_{11}}\right)-A_{4}\\ {\dot{x}}_{5} & = & x_{6}\\ {\dot{x}}_{6} & = & \frac{U_{1}}{m}\left(C_{x_{7}}C_{x_{9}}\right)-g-A_{6}\\ {\dot{x}}_{7} & = & x_{8}\\ {\dot{x}}_{8} & = & \frac{1}{I_{x}}\left(U_{2}+\left(I_{y}-I_{z}\right)x_{10}x_{12}+Jx_{10}\omega \right)-A_{8}\\ {\dot{x}}_{9} & = & x_{10}\\ {\dot{x}}_{10} & = & \frac{1}{I_{y}}\left(U_{3}+\left(I_{z}-I_{x}\right)x_{8}x_{12}+Jx_{8}\omega \right)-A_{10}\\ {\dot{x}}_{11} & = & x_{12}\\ {\dot{x}}_{12} & = & \frac{1}{I_{z}}\left(U_{4}+\left(I_{x}-I_{y}\right)x_{8}x_{10}\right)-A_{12} \end{array}$$

where $A_{2},A_{4},A_{6},A_{8},A_{10},A_{12}$ are unknown but bounded perturbations; $I_{x},I_{y},I_{z}$ are inertial terms; *m* is the mass of the drone; and $\omega =-\omega_{1}+\omega_{2}-\omega_{3}+\omega_{4}$. Moreover, the input vector $U={\left(U_{1},U_{2},U_{3},U_{4}\right)}^{T}$ is composed of

$$\begin{array}{c} \begin{array}{c} U_{1}=F_{1}+F_{2}+F_{3}+F_{4}\\ U_{2}=d\left(F_{4}-F_{2}\right)\\ U_{3}=d\left(F_{3}-F_{1}\right)\\ U_{4}=c\left(-F_{1}+F_{2}-F_{3}+F_{4}\right) \end{array} \end{array}$$

where $F_{i}=bw_{i}^{2},\;\left(i=1,2,3,4\right)$, with *b* as the thrust factor, which is the thrust generated by each rotor. Moreover, *d* is the distance from the center of mass to the rotor, and *c* is the drag factor. For a more comprehensive analysis of the modeling process, please refer to [34]. The Simulink model of the quadrotor mathematical model is shown in Figure 6.

**Figure 7:**
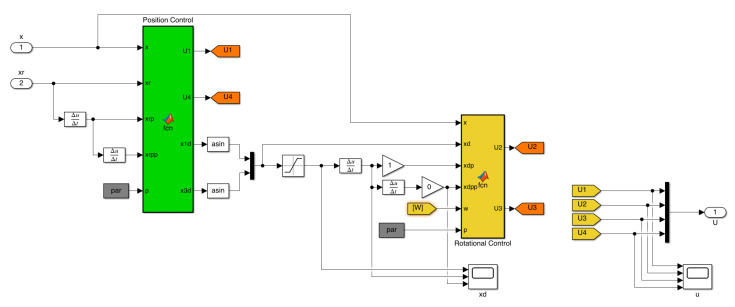
Control scheme for a quadrotor in Simulink.

Moreover, the control subsystem block comprising the drone flight controller can be seen in Figure 6. For this trajectory tracking controller, the control objective is to design the control inputs $U_{1},U_{2},U_{3},U_{4}$ such that the system’s outputs $x_{1},x_{3},x_{5},x_{11}$ track the desired references $x_{1r}\left(t\right),x_{3r}\left(t\right),x_{5r}\left(t\right),x_{11r}\left(t\right)$. Figure 7 depicts the complete trajectory tracking control involving the position and rotational control. The position control has the reference position drone variables $x_{1r}\left(t\right),x_{3r}\left(t\right),x_{5r}\left(t\right)$ as inputs. It generates the desired variables $x_{1d}$ and $x_{3d}$, which serve as input for the rotational control along the reference for yaw angles $x_{11r}$. The complete control input vector is the output for this block.

### 3.4. HIL Simulation in Simulink

The HIL simulation is commonly used to test controller design. It shows the controller’s response in real time to realistic virtual stimuli. In addition, the HIL simulation can also be used to validate a physical system (plant) model.

**Figure 8:**
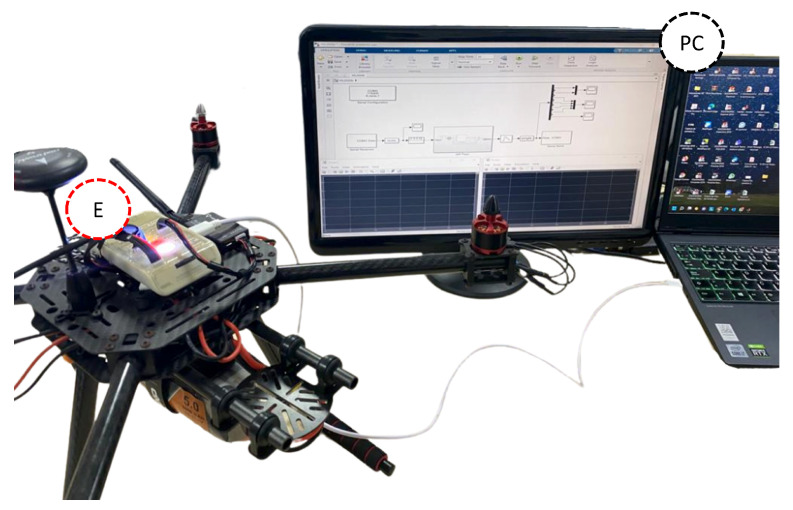
Components of the HIL simulation: the embedded system (E) and the personal computer (PC).

In this HIL simulation, a real-time computer is used for the virtual representation of the UAV plant model and an embedded system as a real version of the UAV flight controller (see Figure 8). The embedded system (development hardware) is the RDDRONE-FMUK66 vehicle/flight management unit (FMU), which is supported by the business-friendly open source PX4.org (accessed on 1 July 2022) flight stack. It is worth mentioning that the embedded system is part of the NXP HoverGames drone kit (KIT-HGDRONEK66).

**Figure 9:**
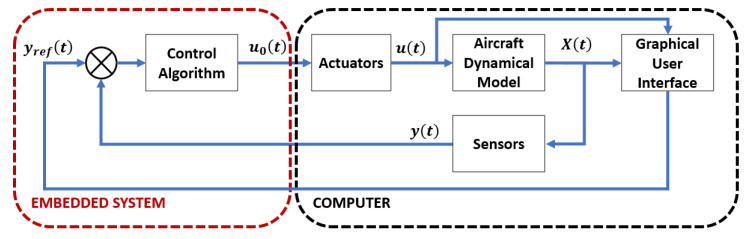
Overall proposed hardware-in-the-loop simulation architecture.

The proposed HIL architecture is shown in Figure 9. HIL testing simulates the drone variables collected by the sensors and the reference signals and sends them to the FMU being tested, making it believe that it is reacting to real-world flight conditions. The HIL simulation contains all the relevant components of the drone. The HIL simulation approach supports the verification and validation activities.

## 4. Instructional Design for Drone Flight Basics within the EMCF

The quadrotor is an aerial robot useful when dealing with several concepts such as translation, rotation, line segmentation, and path planning, among other topics. This work considers the teaching case for which the instructional design is devoted to constructing the mechatronic concept of drone navigation by waypoints under the EMCF, involving the perspective entities: Dynamics (process) + Robotics (Application) + Drone (Artifact). Then, the pedagogical activities for the three levels with the selected perspective are developed in the following subsections. It is worthwhile to mention that the three basic movements when starting drone flight considered in this work are:

1. Forward–backward movement;
2. Plus sign movement;
3. Square array movement.

To start the practice, the instructor turns on the MoCap system, places the drone matching the origin of the MoCap with zero Euler angles, and turns it on the drone and its remote controller. Then, they start tracking the drone object with Vicon Tracker and open Simulink to start plotting the 3D graph for the participant.

### 4.1. Concrete Level (First Learning Construction Level)

In this level, one must design activities oriented to perceptuo-motor characteristics. Here, a drone, DJI Phantom 4, is chosen in order to provide the participant with the experience of flying a drone, starting with a real flight in a real environment. The designed activities are possible thanks to the factory’s controller that the drone comes with, which is a speed manual control. If the participant does not move the remote control sticks, the drone will remain in the same place, and only move when the remote controller sticks are moved in any direction.

**Figure 10:**
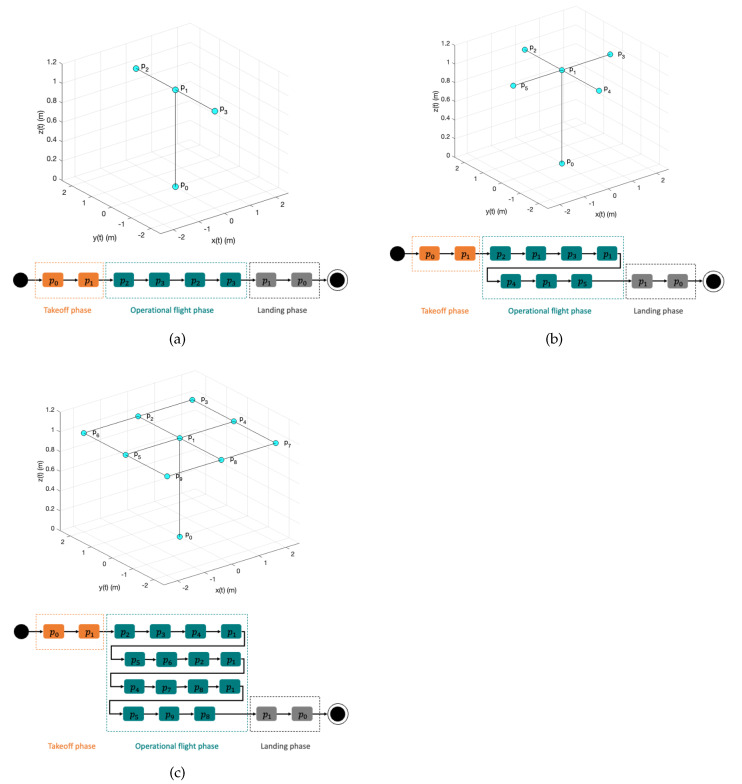
Concrete-level movements. (**a**) First movement: forward–backward. (**b**) Second movement: plus sign. (**c**) Third movement: square array.

First, the flight plan for the first movement is given to the participant (see Figure 10a); it includes the state diagram showing the sequence in which the pilot must reach each waypoint.

Then, the instructor must start recording the position and orientation data. The set of instructions for participants are the following; it is worthwhile to mention that the drone starts in its home position $P_{0}=\left(x,y,z\right)=\left(0,0,0\right)$.

- The takeoff phase involves two steps: turning the motors on and elevating the drone to a specific altitude:
  1. Raise left stick up slowly to take off until the drone reaches approximately 1 m; then, return the left stick to its center position slowly. Note: Left stick controls height (up–down) and heading (left–right). The drone reaches the waypoint $P_{1}=\left(0,0,1\right)$.
- The operational flight phase involves three steps: move forward, move backward, and repeat the process:
  2. Raise right stick up slowly to move the drone forward until it reaches approximately 2 m; then, return the right stick to its center position slowly. The drone reaches the waypoint $P_{2}=\left(0,2,1\right)$.
  3. Lower right stick down slowly to move the drone backward until it reaches approximately −2 m; then, return the right stick to its center position slowly. Here, the drone reaches the waypoint $P_{3}=\left(0,-2,1\right)$.
  4. Repeat the process twice and return to the center position, $P_{1}=\left(0,0,1\right)$, where we started the previous phase. Now, we are ready to start the landing phase. Note: The right stick controls forward, backward, left, and right movements.
- The landing phase involves one step:
  5. Lower left stick down slowly until the drone touches the ground and hold it for a few seconds to stop the motors. Then, the drone reaches its home position again $P_{0}=\left(0,0,0\right)$.
  (Instruction remark: the instructor stops recording the data. The MoCap system records the position and orientation measurements of the drone in an Excel file. This table will contain the set of points that capture the real movement of the drone and it can be found in https://acortar.link/Rb54SB (accessed on 1 July 2022).

Once the pilot finishes the first movement, he/she continues with the second and third movements. The flight plans, including the state diagram showing the sequence in which the pilot must reach each waypoint for these movements, are shown in Figure 10b,c, respectively.

**Figure 11:**
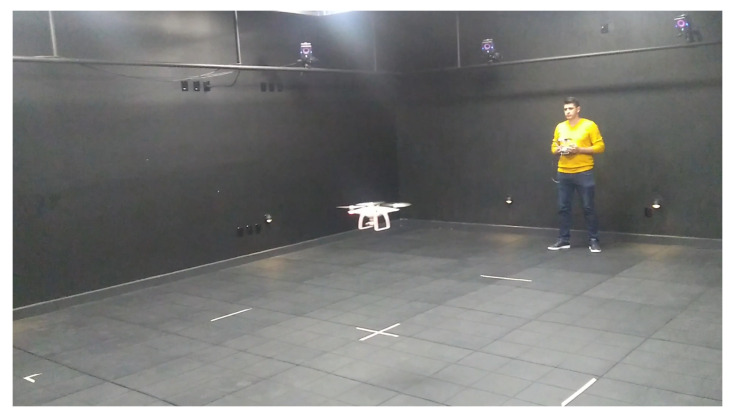
Drone flight in the concrete level.

In addition, Figure 11 shows the pilot performing the flights in the MoCap laboratory. Moreover, the video showing the drone pilot performing the flights according to the plans and the instructions in this level can be found at https://acortar.link/yE0nKw (accessed on 1 July 2022).

### 4.2. Graphic Level (Second Learning Construction Level)

In this level, one must design activities oriented to graphic (symbolic) representation of the mechatronic concept, taking as a reference the previously developed concept at the concrete learning level; this will allow us to gradually make the transition from concrete to abstract. The Excel file containing the recorded data and a program in Simulink are given to the participant to plot it. In addition, this level allows dynamic color changes of the virtual images (such as circles or squares in a dynamic manner) but without allowing the further movement of the drone [35].

The set of instructions for participants is as follows.

- The takeoff phase involves two positions:
  1. Draw an orange vertical dotted line in the position vector plot for representing the drone home position $P_{0}$, in time t=0 s, and label it at the top of the graph.
  2. Then, draw a blue round dot line representing the drone reference waypoint $P_{1r}$ in which the *z* position starts to increase its value, in time t=3.5 s. Label it at the bottom of the graph.
  3. Now, draw another orange vertical dotted line when the drone reaches the waypoint $P_{1}$, in time t=5 s. Label it at the top of the graph.
  4. Draw a blue round dot line representing the next drone reference waypoint $P_{2r}$ in which the *y* position starts to increase its value, in time t=6 s. Label it at the bottom of the graph.
  5. Finally, draw a filled orange rectangle from $P_{0}$ to $P_{2r}$. (Instruction remark: This rectangle encompasses the drone takeoff phase).
- The operational flight phase involves four positions:
  6. Draw a green vertical dotted line when the drone reaches the waypoint $P_{2}$, in time t=8 s. Label it at the top of the graph.
  7. Draw a blue round dot line representing the next drone reference waypoint $P_{3r}$ in which the *y* position starts to decrease its value, in time t=9 s. Label it at the bottom of the graph.
  8. Draw a green vertical dotted line when the drone reaches the waypoint $P_{3}$, in time t=15 s. Label it at the top of the graph.
  9. Draw a blue round dot line representing the next drone reference waypoint $P_{2r}$ in which the *y* position starts to increase its value, in time t=16 s. Label it at the bottom of the graph.
  10. Draw a green vertical dotted line when the drone reaches the waypoint $P_{2}$, in time t=19 s. Label it at the top of the graph.
  11. Draw a blue round dot line representing the next drone reference waypoint $P_{3r}$ in which the *y* position starts to decrease its value, in time t=21 s. Label it at the bottom of the graph.
  12. Draw a green vertical dotted line when the drone reaches the waypoint $P_{3}$, in time t=25.5 s. Label it at the top of the graph.
  13. Draw a blue round dot line representing the next drone reference waypoint $P_{1r}$ in which the *y* position starts to increase its value, in time t=26 s. Label it at the bottom of the graph.
  14. Finally, draw a filled green rectangle from $P_{2r}$ in t=16 s to $P_{1r}$ in t=26 s. (Instruction remark: This rectangle encompasses the drone operational flight phase).
- The landing phase involves one step:
  15. Draw a gray vertical dotted line when the drone reaches the waypoint $P_{1}$, in time t=28 s. Label it at the top of the graph.
  16. Draw a blue round dot line representing the next drone reference waypoint $P_{0r}$ in which the *z* position starts to decrease its value, in time t=29 s. Label it at the bottom of the graph.
  17. Draw a gray vertical dotted line when the drone reaches the waypoint $P_{0}$, in time t=33 s. Label it at the top of the graph.
  18. Finally, draw a filled gray rectangle from $P_{1r}$ in t=26 s to the last recorded datum corresponding to the home position $P_{0}$. (Instruction remark: This rectangle encompasses the drone landing phase).

**Figure 12:**
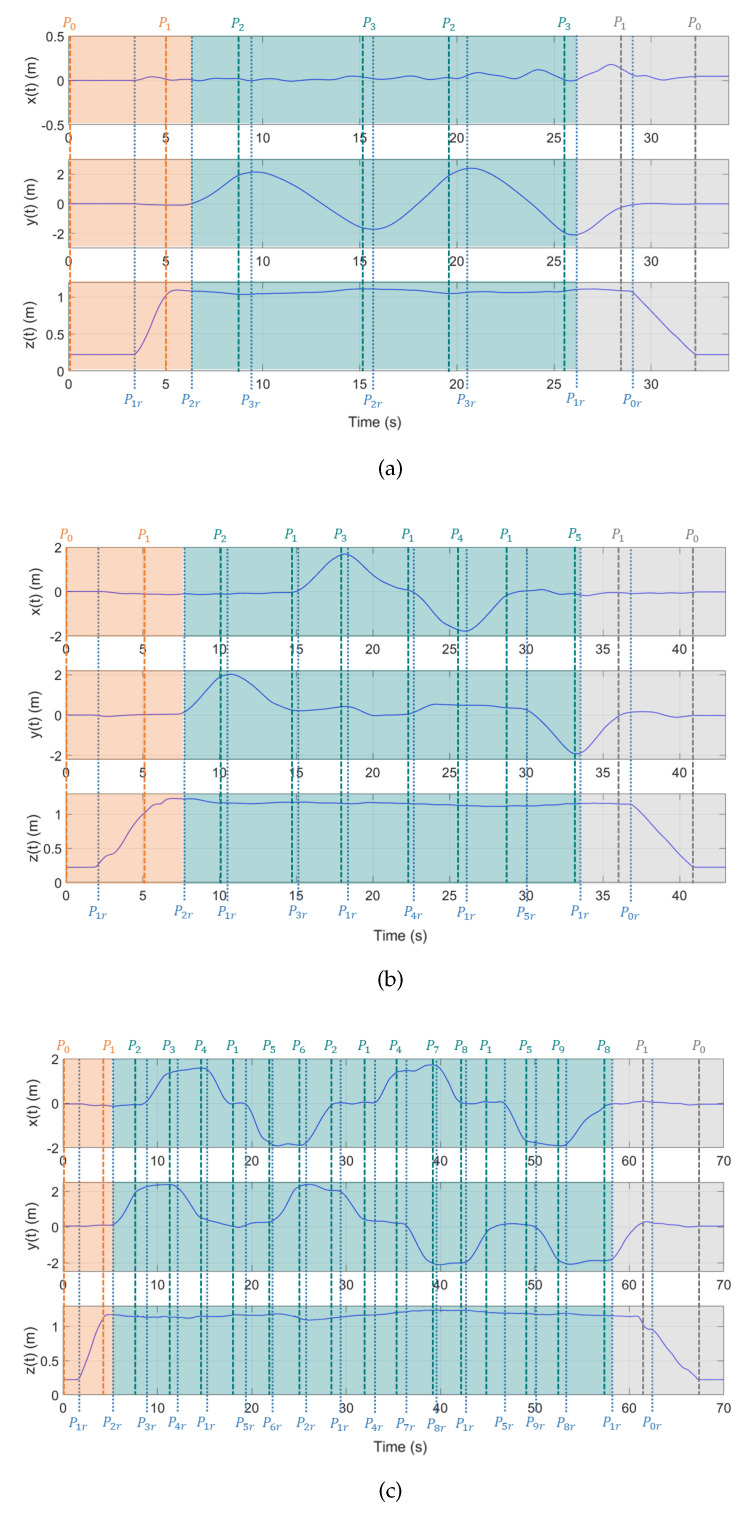
Graphic-level results. (**a**) Graphic-level results of the 1st movement. (**b**) Graphic-level results of the 2nd movement. (**c**) Graphic-level results of the 3rd movement.

The resulting graph when applying the graphic level is shown in Figure 12a. Once the pilot finishes the first movement, he/she continues with the second and third movements. The obtained graphs for these movements are shown in Figure 12b,c, respectively.

### 4.3. Abstract Level (Third Learning Construction Level)

This level involves designing activities oriented towards gradually transitioning from the graphic (symbolic) concepts to a more abstract representation. The drone navigation for the first movement defined by waypoints can be seen in Figure 12a. Reference waypoints appear at the bottom of the graph, which will be used in the simulation in Simulink to test the mathematical model and the trajectory control of the drone.

The set of instructions for the participant is as follows.

- Build the pseudocode of the waypoint generation for the 1st movement. First, define as the input all the reference waypoints that appear in Figure 12a, i.e., $P_{0r}=\left(0;0;0\right),P_{1r}=\left(0;0;1\right),P_{2r}=\left(0;2;1\right),P_{3r}=\left(0;-2;1\right)$. Then, define the output as the waypoint reference vector $wp_{r}=\left(x_{r},y_{r},z_{r}\right)$.
- Now, introduce a conditional clause **if**, from the time greater than or equal to 0 s to a time before the first reference waypoint $P_{1r}$ will occur, i.e., t=3.5 s; **then,** the reference waypoint vector will be equal to $P_{0}$, the drone’s home position.
- Now, **if** time is greater than or equal to 3.5 s to a time before the second reference waypoint $P_{2r}$ will occur, i.e., t=6 s, **then** the reference waypoint vector will be equal to $P_{1r}$.
- **If** time is greater than or equal to 6 s to a time before the third reference waypoint $P_{3r}$ will occur, i.e., t=9 s, **then** the reference waypoint vector will be equal to $P_{2r}$.
- **If** time is greater than or equal to 9 s to a time before the fourth reference waypoint $P_{2r}$ will occur, i.e., t=16 s, **then** the reference waypoint vector will be equal to $P_{3r}$.
- **If** time is greater than or equal to 16 s to a time before the fifth reference waypoint $P_{3r}$ will occur, i.e., t=21 s, **then** the reference waypoint vector will be equal to $P_{2r}$.
- **If** time is greater than or equal to 21 s to a time before the sixth reference waypoint $P_{1r}$ will occur, i.e., t=26 s, **then** the reference waypoint vector will be equal to $P_{3r}$.
- **If** time is greater than or equal to 26 s to a time before the seventh reference waypoint $P_{0r}$ will occur, i.e., t=29 s, **then** the reference waypoint vector will be equal to $P_{1r}$.
- Finally, **else** the eighth reference waypoint $P_{0r}$ will occur. (Instruction remark: The reference waypoint vector establishes the desired drone trajectory, which encompasses the desired waypoint in space that the drone needs to go though. It is worth mentioning that the actual waypoints $P_{i},i=0,1,2,3$ are reached after the corresponding reference waypoint vector is supplied to the controller in the simulation).

**Figure 13:**
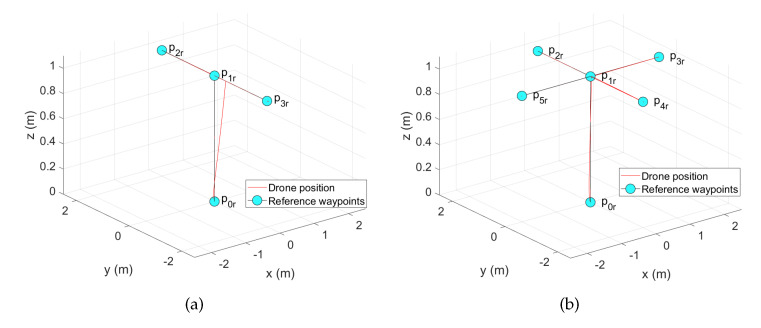

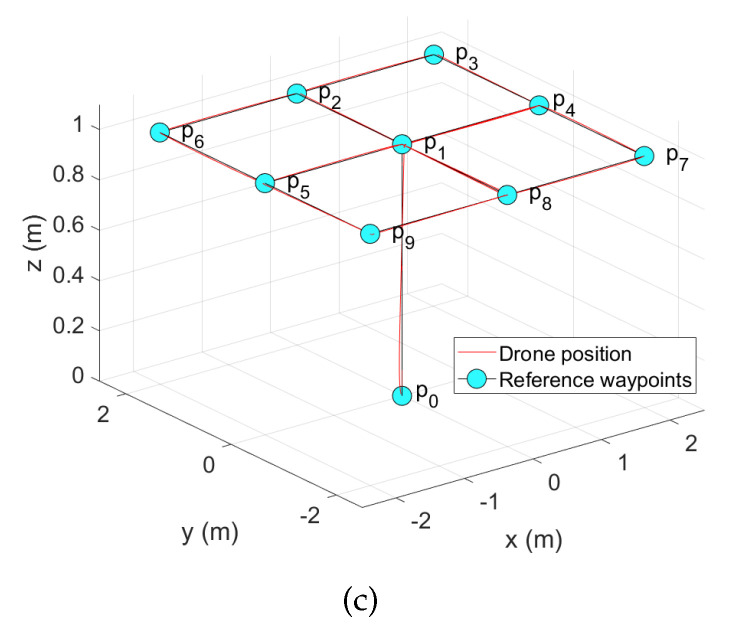
Drone’s absolute position obtained with the simulation in Simulink. (**a**) First movement trajectory tracking with the simulated model of the UAV. (**b**) Second movement trajectory tracking with the simulated model of the UAV. (**c**) Third movement trajectory tracking with the simulated model of the UAV.

The complete pseudocode for the reference waypoint vector can be seen in Algorithm 1. Then, this pseudocode is programmed in a MATLAB file inside the waypoint reference block in the simulation of Figure 6. It is worth mentioning that the obtained behavior, shown in Figure 13, is similar to the graph in Figure 12. The participant can see the mathematical model’s importance for future works. Once the pilot finishes the first movement, he/she continues with the second and third movements. The obtained pseudocodes for these movements are shown in Algorithms 2 and 3, respectively.
 **Algorithm 1** Waypoint generation for the 1st movement**Input: ***Waypoints*: $P_{0r}=\left(0;0;0\right),P_{1r}=\left(0;0;1\right),P_{2r}=\left(0;2;1\right),P_{3r}=\left(0;-2;1\right)$**Output:** *Waypoint reference vector*: $wp_{r}=\left(x_{r},y_{r},z_{r}\right)$     1:**if** time ≥0 and time <3.5 **then**     2:    $wp_{r}=P_{0}$     3:**end if**     4:**if** time ≥3.5 and time <6 **then**     5:    $wp_{r}=P_{1r}$     6:**end if**     7:**if** time ≥6 and time <9 **then**     8:    $wp_{r}=P_{2r}$     9:**end if**     10:**if** time ≥9 and time <16 **then**     11:    $wp_{r}=P_{3r}$     12:**end if**     13:**if** time ≥16 and time <21 **then**     14:    $wp_{r}=P_{2r}$     15:**end if**     16:**if** time ≥21 and time <26 **then**     17:    $wp_{r}=P_{3r}$     18:**end if**     19:**if** time ≥26 and time <29 **then**     20:    $wp_{r}=P_{1r}$     21:**else**     22:    $wp_{r}=P_{0r}$     23:**end if**

Moreover, the HIL simulation strengthens the assimilation of the UAV flight dynamics since it permits the interaction between a simulated model of the UAV and the digital implementation of its automatic control algorithm (see Algorithm 4 and Figure 7).

**Figure 14:**
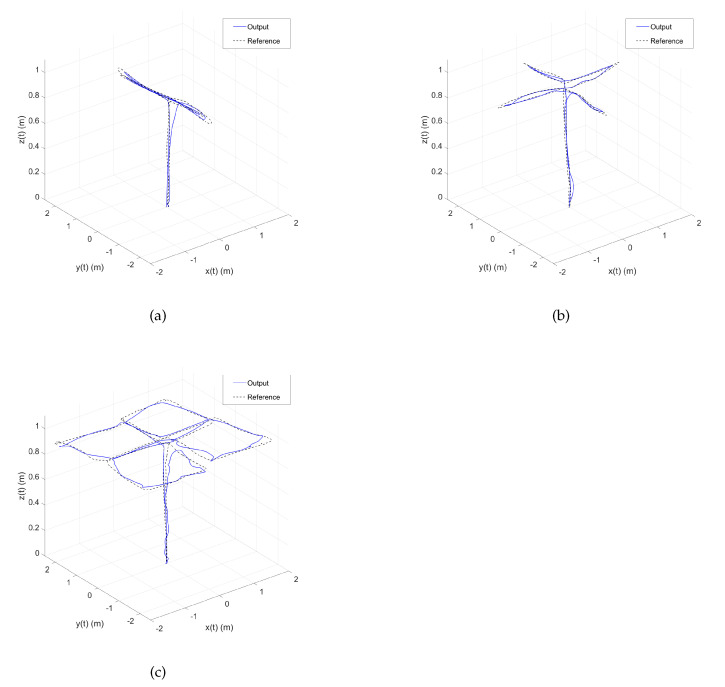
Drone’s absolute position obtained with the HIL simulation in Simulink. (**a**) First movement trajectory tracking with the HIL simulated model. (**b**) Second movement trajectory tracking with the HIL simulated model. (**c**) Third movement trajectory tracking with the HIL simulated model.

The obtained behavior is similar to the real flight and the simulation model of the UAV. Figure 14 depicts the overall proposed HIL simulation results.
 **Algorithm 2** Waypoint generation for the 2nd movement**Input:** *Waypoints*: $P_{0r}=\left(0;0;0\right),P_{1r}=\left(0;0;1\right),P_{2r}=\left(0;2;1\right),P_{3r}=\left(2;0;1\right),P_{4r}=\left(-2;0;1\right),P_{5r}=\left(0;-2;1\right)$**Output: ***Waypoint reference vector*: $wp_{r}=\left(x_{r},y_{r},z_{r}\right)$     1:**if** time ≥0 and time <2 **then**     2:    $wp_{r}=P_{0}$     3:**end if**     4:**if** time ≥2 and time <7.5 **then**     5:    $wp_{r}=P_{1r}$     6:**end if**     7:**if** time ≥7.5 and time <10.5 **then**     8:    $wp_{r}=P_{2r}$     9:**end if**     10:**if** time ≥10.5 and time <15 **then**     11:    $wp_{r}=P_{1r}$     12:**end if**     13:**if** time ≥15 and time <18 **then**     14:    $wp_{r}=P_{3r}$     15:**end if**     16:**if** time ≥18 and time <22.5 **then**     17:    $wp_{r}=P_{1r}$     18:**end if**     19:**if** time ≥22.5 and time <26 **then**     20:    $wp_{r}=P_{4r}$     21:**end if**     22:**if** time ≥26 and time <30 **then**     23:    $wp_{r}=P_{1r}$     24:**end if**     25:**if** time ≥30 and time <34 **then**     26:    $wp_{r}=P_{5r}$     27:**end if**     28:**if** time ≥34 and time <37 **then**     29:    $wp_{r}=P_{1r}$     30:**else**     31:    $wp_{r}=P_{0r}$     32:**end if**

This instructional design is devoted to boosting the development and construction of the mechatronic concept of drone navigation by waypoints. Here, a waypoint is an intermediate point on a drone’s route or line of travel. Drone navigation by waypoints allows a drone to fly with its flying points preplanned; thus, we know exactly where the drone needs to go directly for its first point and can proceed to next point until we complete these preplanned sequences. It is worthwhile to mention that developing the knowledge, skills, and attitudes of the new personnel responsible for the new jobs generated by Industry 4.0 is key since new robot configurations, controllers, sensors, and devices will be required. Therefore, this drone flight educational mechatronics system based on the EMCF is oriented towards helping in this imminent technological transition.
 **Algorithm 3** Waypoint generation for the 3rd movement**Input:** *Waypoints*: $P_{0r}=\left(0;0;0\right),P_{1r}=\left(0;0;1\right),P_{2r}=\left(0;2;1\right),P_{3r}=\left(2;2;1\right),P_{4r}=\left(2;0;1\right),P_{5r}=\left(-2;0;1\right),P_{6r}=\left(-2;2;1\right),P_{7r}=\left(2;-2;1\right),P_{8r}=\left(0;-2;1\right),P_{9r}=\left(-2;-2;1\right)$**Output:** *Waypoint reference vector*: $wp_{r}=\left(x_{r},y_{r},z_{r}\right)$1:**if** time ≥0 and time <2 **then**2:    $wp_{r}=P_{0}$3:**end if**4:**if** time ≥2 and time <5 **then**5:    $wp_{r}=P_{1r}$6:**end if**7:**if** time ≥5 and time <8 **then**8:    $wp_{r}=P_{2r}$9:**end if**10:**if** time ≥8 and time <12 **then**11:    $wp_{r}=P_{3r}$12:**end if**13:**if** time ≥12 and time <15 **then**14:    $wp_{r}=P_{4r}$15:**end if**16:**if** time ≥15 and time <19 **then**17:    $wp_{r}=P_{1r}$18:**end if**19:**if** time ≥19 and time <22 **then**20:    $wp_{r}=P_{5r}$21:**end if**22:**if** time ≥22 and time <25 **then**23:    $wp_{r}=P_{6r}$24:**end if**25:**if** time ≥25 and time <29 **then**26:    $wp_{r}=P_{2r}$27:**end if**28:**if** time ≥29 and time <33 **then**29:    $wp_{r}=P_{1r}$30:**end if**31:**if** time ≥33 and time <36 **then**32:    $wp_{r}=P_{4r}$33:**end if**34:**if** time ≥36 and time <39 **then**35:    $wp_{r}=P_{7r}$36:**end if**37:**if** time ≥39 and time <43 **then**38:    $wp_{r}=P_{8r}$39:**end if**40:**if** time ≥43 and time <46 **then**41:    $wp_{r}=P_{1r}$42:**end if**43:**if** time ≥46 and time <50 **then**44:    $wp_{r}=P_{5r}$45:**end if**46:**if** time ≥50 and time <54 **then**47:    $wp_{r}=P_{9r}$48:**end if**49:**if** time ≥54 and time <58 **then**50:    $wp_{r}=P_{8r}$51:**end if**52:**if** time ≥58 and time <61 **then**53:    $wp_{r}=P_{1r}$54:**else**55:    $wp_{r}=P_{0r}$56:**end if**

 **Algorithm 4** Embedded control for HIL simulation
**Input:** *References*: $y_{ref}$=($x_{5r}$, $x_{7r}$, $x_{9r}$, $x_{11r}$)${}^{T}$,
   *States*: $X={\left[x,\dot{x},y,\dot{y},z,\dot{z},\phi ,\dot{\phi },\theta ,\dot{\theta },\psi ,\dot{\psi }\right]}^{T}$,
   *Time*: *t*.
**Output:** *Control forces*: $U={\left(U_{1},\;U_{2},\;U_{3},\;U_{4}\right)}^{T}$
     1:$t_{a}\leftarrow t$                                             ▹ Initial time     2:**while** run **do**     3:    $U_{4}\leftarrow yaw\_control\left(X,x_{5r}\right)$     4:    $U_{1}\leftarrow altitude\_control\left(X,x_{7r}\right)$     5:    $x_{1d}\leftarrow longitude\_control\left(X,x_{9r},x_{11r}\right)$     6:    $x_{3d}\leftarrow latitude\_control\left(X,x_{9r},x_{11r}\right)$     7:    $U_{2}\leftarrow roll\_control\left(X,x_{1d}\right)$     8:    $U_{3}\leftarrow pitch\_control\left(X,x_{3d}\right)$     9:    **if** $t-t_{a}<10\mathrm{ms}$ **then**     10:        wait(10-t+$t_{a}$) ▹ Time in milliseconds     11:    **end if**     12:    $t_{a}\leftarrow t$     13:
**end while**



## 5. Discussion

The application of UAVs in this work is not a coincidence, as recent studies present the need to integrate unmanned aerial vehicle (UAV) training into STEAM education [36,37]. UAVs have been widely used in the science, technology, engineering, arts, and mathematics (STEAM) areas, giving the students a wide range of uses in the STEAM fields and teaching them a set of valuable skills and abilities, such as the work in [9].

As mentioned in the Introduction, hardware-in-the-loop (HIL) simulation is an essential approach in the fields of autonomous vehicles and robotics. It is widely used in the automotive industry, among others. HIL helps to accelerate the design and testing phases in engineering. This work has integrated a complete solution—the UAV-based smart educational mechatronics system using a MoCap laboratory and HIL—within an educational framework, namely the EMCF. This system represents a great advantage to the academic and industrial environment as students can quickly appropriate and integrate new technologies within extensively used mechatronic concepts. The developed system opens up a new range of possibilities in terms of knowledge construction through instructional designs for practices, activities, and tasks, enriching the university courses.

Table 1 shows the context in which this work is developed and the trend toward including new and more educational tools in pursuing new educational experiences.

The market for robots and drones has increased exponentially in recent years. This will have a significant influence in the long term due to the transformation of industry features in many areas, such as agriculture, logistics, cleaning, and more. The market is estimated to grow by nearly 3 and 7 times in the next 10 and 20 years, respectively [38].

Drones/UAVs have seen many improvements in a number of aspects, such as geometric structure, flying mechanism, sensing and vision ability, aviation quality, path planning, intelligent behavior, and adaptability [39]. All these features are essential to understanding the importance of subsequent development. In particular, robot navigation remains a fundamental topic within the robotics research field. Although technological advances allow us to reduce the learning curve and appropriation of these new technologies, it is crucial to increase the levels of abstraction, to more closely resemble how humans navigate and perform tasks in different environments.

**Table 1:** Comparison between related works using educational tools: drone, MoCap, and HIL simulation.

Educational Tools	Scientific Articles
Drone	[9,36,40,41,42]
Drone + MoCap	[43,44,45]
Drone + HIL simulation	[46,47,48]
Drone + MoCap + HIL simulation	Present work

The presented instructional design represents a useful tool to introduce the actors of the engineering world to the technological transition of Industry 4.0. However, some important stages addressed in most of the current technological developments are missed: model-based design and testing of algorithms, simulation alternatives, and digital implementations. These fields represent opportunities for future work in the current instructional design to enhance its capabilities, applicability, and versatility. On the other hand, comparing other educational methodologies and their respective performance and results in real-time experiments would also be valuable.

## 6. Conclusions

The instructional design for drone navigation by waypoints is intended to better prepare students for drone flight to acquire the necessary knowledge for using these smart devices for applications in Industry 4.0. The developed UAV-based smart educational mechatronics system using a MoCap laboratory and HIL represents an effort toward an educational concept focused on equipping students with the knowledge and skills required to meet the new demands of companies.

The system’s main features include the use of all the components within the EMCF, including the HIL simulation. Its main functionalities are validating a physical drone model and testing existing or new control algorithms without putting the real drone at risk. These allow the development of UAV knowledge and skills.

We consider it vital to disseminate educational mechatronics to help countries to transform into relevant actors in the fourth industrial revolution. In future work, this instructional design is planned to be applied to engineering students. However, the pandemic has prevented us from implementing it; we hope that this will change shortly. Moreover, it is worthwhile to mention that evaluating the flights’ performance and considering sensors for outdoor environments with obstacles are considered the next steps.

## Data Availability

Data supporting the reported results can be found at https://acortar.link/Rb54SB (accessed on 1 July 2022).

## Abbreviations

The following abbreviations are used in this manuscript:

UAV	Unmanned Aerial Vehicle
MoCap	Motion Capture
HIL	Hardware-in-the-Loop
EMCF	Educational Mechatronics Conceptual Framework
AI	Artificial Intelligence
IoT	Internet of Things
ICT	Information and Communications Technology
STEAM	Science, Technology, Engineering, Arts, and Mathematics
PBL	Problem-Based Learning
PoE	Power over Ethernet
FMU	Flight Management Unit
PC	Personal Computer
